# Buffalo Medical College

**Published:** 1851-06

**Authors:** 


*Buffalo Medical College.*—Some changes have recently taken place in the organization of this Institution. Prof. B. R. Palmer, of Woodstock, Vt., has been appointed to the chair of Anatomy, vacated by the resignation of Prof. Webster announced in a former number of this Journal.

Prof. C. B. Coventry, having tendered his resignation, has been appointed "in consideration of his services in behalf of the College, and as a token of the high respect in which he is held by the Council and Medical Faculty," Emeritus Professor of Physiology and Medical Jurisprudence. Dr. John C. Dalton, jr., of Boston, Mass., has been appointed to the chair of Physiology and Medical Jurisprudence, vacated by the resignation of Prof. Coventry.

The new appointments will, it is believed, be satisfactory to the friends of the College. Prof. Palmer is too well known as an eminent teacher of anatomy to require aught to be said in his behalf. Prof. Dalton, although, comparatively, a young man, is already distinguished for his physiological and pathological researches. It will be observed by reference to another article in the present No., that to Dr. D. has been awarded the prize offered by the American Medical Association for the best "*experimental* essay on a subject connected either with Physiology or Medical Chemistry."

